# Effectiveness of a Self-Guided Web-Based Cannabis Treatment Program: Randomized Controlled Trial

**DOI:** 10.2196/jmir.2256

**Published:** 2013-02-15

**Authors:** Sally Rooke, Jan Copeland, Melissa Norberg, Donald Hine, Jim McCambridge

**Affiliations:** ^1^University of New South WalesRandwickAustralia; ^2^University of New EnglandArmidaleAustralia; ^3^London School of Hygiene and Tropical MedicineLondonUnited Kingdom

**Keywords:** marijuana, Internet intervention, computer-assisted therapy, addiction, randomized controlled trial

## Abstract

**Background:**

Self-help strategies offer a promising way to address problems with access to and stigma associated with face-to-face drug and alcohol treatment, and the Internet provides an excellent delivery mode for such strategies**.** To date, no study has tested the effectiveness of a fully self-guided web-based treatment for cannabis use and related problems.

**Objectives:**

The current study was a two-armed randomized controlled trial aimed at testing the effectiveness of *Reduce Your Use*, a fully self-guided web-based treatment program for cannabis use disorder consisting of 6 modules based on cognitive, motivational, and behavioral principles.

**Methods:**

225 individuals who wanted to cease or reduce their cannabis use were recruited using both online and offline advertising methods and were randomly assigned to receive: (1) the web-based intervention, or (2) a control condition consisting of 6 modules of web-based educational information on cannabis. Assessments of cannabis use, dependence symptoms, and abuse symptoms were conducted through online questionnaires at baseline, and at 6-week and 3-month follow-ups. Two sets of data analyses were undertaken—complier average causal effect (CACE) modeling and intention to treat (ITT).

**Results:**

Two thirds (149) of the participants completed the 6-week postintervention assessment, while 122 (54%) completed the 3-month follow-up assessment. Participants in the intervention group completed an average of 3.5 of the 6 modules. The CACE analysis revealed that at 6 weeks, the experimental group reported significantly fewer days of cannabis use during the past month (*P*=.02), significantly lower past-month quantity of cannabis use (*P*=.01), and significantly fewer symptoms of cannabis abuse (*P*=.047) relative to controls. Cannabis dependence symptoms (number and severity) and past-month abstinence did not differ significantly between groups (*P*s>.05). Findings at 3 months were similar, except that the experimental group reported significantly fewer and less severe cannabis dependence symptoms (*P*s<.05), and past-month quantity of cannabis consumed no longer differed significantly between groups (*P*=.16). ITT analyses yielded similar outcomes.

**Conclusion:**

Findings suggest that web-based interventions may be an effective means of treating uncomplicated cannabis use and related problems and reducing the public health burden of cannabis use disorders.

**Trial registration:**

ACTRN12609000856213, Australian New Zealand Clinical Trials Registry.

## Introduction

Cannabis is the most commonly used illicit drug in the developed world—1.9 million Australians, for example, reported using cannabis in 2010 [1]. Although once believed to be a relatively harmless substance, it is now known that approximately one out of 10 of those who ever use cannabis meet DSM-IV criteria for cannabis dependence at some point in time [2]. Heavy cannabis use is associated with poorer mental and physical health, lower educational achievement, and impoverished cognitive functioning [3]. In Australia, cannabis use accounted for 10% of the health burden relating to all illicit drug use in 2003, with only heroin and hepatitis C contributing more [4]. This entails a greater loss of disability-adjusted healthy life years than the loss attributed to suicide and self-inflicted injuries related to substance use. Other developed countries, such as the United States, have experienced similar rates of cannabis use and related problems [5].

Unfortunately, most individuals meeting diagnostic criteria for a cannabis use disorder do not seek professional treatment [2]. This can occur for a variety of reasons. First, many cannabis users are employed and unable to attend face-to-face sessions during working hours. Second, residents of remote areas or localities poorly serviced by public transport have difficulty traveling to regular sessions. Third, face-to-face therapy is economically burdensome and provision services frequently cannot meet demand [6]. Finally, many people hesitate to seek treatment due to concerns about confidentiality and being stigmatized [7]. These issues underscore the vital need for evidence-based treatments that are highly accessible, financially efficient, and have a high level of acceptability to consumers.

Internet-delivered treatments may assist in resolving these issues, offering several advantages, including bridging the gap between supply and demand for alcohol and drug therapists, being potentially more cost effective than face-to-face treatment, and having the ability to be accessed at most times and in most locations. Increased privacy largely addresses the issue of stigmatization. Additionally, where treatment is automated, it is consistently delivered in its intended manner [8].

Several computer programs and web-based interventions for substance use have recently been developed and tested for their efficacy. The treatments consist of components such as cognitive behavioral therapy (CBT) [9], chat forums [10], and normative feedback on substance use [11]. A recent meta-analysis of the efficacy of computer-delivered treatments for tobacco and alcohol use found that, overall, the treatments had a significant effect [12]. A nonrandomized study by Budney et al involving 38 participants found that a computerized intervention program with therapist support yielded similar reductions in cannabis use to a therapist-delivered intervention [13]. Tossmann et al tested the effects of a therapist-assisted online treatment program for cannabis use in a randomized trial with high levels of attrition, finding significant effects on cannabis use reductions [14]. Sinadinovic et al found some evidence that an online brief intervention program was superior to assessment-only in assisting illicit drug users to reduce their substance use [15]. No previous study, however, has empirically tested the efficacy of a fully self-guided web-based treatment for cannabis use and related problems.

### Research Objectives and Hypotheses

In response to the absence of evidence-based fully self-guided online treatments for cannabis use, the authors developed the online program, *Reduce Your Use: How to Break the Cannabis Habit* [16]. The objective of the current study was to test the effectiveness of the program in assisting individuals who wished to reduce or stop their cannabis use. We hypothesized that at 6-week and 3-month follow-up assessments, relative to an information-only control group, individuals who were randomized to the intervention would report lower frequency of cannabis use (H1), lower quantity of cannabis use (H2), lower levels of cannabis dependence (H3), and fewer symptoms of cannabis abuse (H4). We further hypothesized that the intervention group would report higher rates of past-month abstinence at both follow-up points (H5).

## Methods

### Participants

Our power calculation was based on a projected effect size of *d*=0.45, as this was obtained for cannabis use frequency in the face-to-face treatment on which the intervention was based [17]. This required a total of 158 participants to achieve 80% power. However, given that web-based studies are prone to higher attrition rates than face-to-face treatments [18], we recruited a larger number of participants (N=225). Participants were recruited between April 2010 and May 2011.

Participants were primarily from Australia (64%); however, Google advertising also attracted participants from the United Kingdom (21%), the United States (10%), New Zealand (3%), and other countries (2%). Study participants were adults who were at least 18 years old and were English and computer literate in order to comply with study procedures. All participants had used cannabis at least once during the past month and expressed a desire to stop or reduce their cannabis use. Those who had received formal treatment for cannabis use or any other substance use within the past 3 months were excluded from the study, as were those who used another illicit drug weekly or more frequently, or who reported having a mental illness that would be likely to significantly interfere with their participation. This information was obtained first by asking the participant if they had any mental illness that would likely interfere with their participation, then by asking them if a doctor had diagnosed them with schizophrenia, schizoaffective disorder, or bi-polar disorder. Participants who answered yes to either of these questions were excluded from participation.

### Procedures

Ethical approval for this study was given by the University of New South Wales (UNSW) Human Research Ethics Committee. Approval was granted to recruit participants both within Australia and elsewhere. Recruitment for the study commenced following in-house testing of the program, during which minor modifications were made and bugs were fixed. Advertisements seeking individuals who wished to reduce or quit their cannabis use via an online program were placed on the National Cannabis Prevention and Information Centre (NCPIC) website, online forums, Google, university bulletin boards, in newspapers, and at community health centers. NCPIC and UNSW affiliations were displayed on all advertisements. Interested individuals contacted the research team via email and were sent screening and study information materials by return email. Inclusion/exclusion criteria (aside from being 18 or older) were not stated on the advertisement, nor specifically noted during participant screening, to prevent individuals from providing false information in order to be eligible for the study. Compensation for completing assessments was not noted in the study advertisement but was noted in the participant information sheet, which participants received after contacting us to indicate their interest in the study.

Participants were informed that they would be randomly assigned to receive 6 modules of CBT or 6 modules of educational information. After responding to the screening questions and prior to completing the baseline assessment, those eligible for participation were randomly assigned by the first author. Assignment occurred through the drawing of one of two tokens from a box. The tokens were two different colours, representing the two study conditions. The token was replaced each time it was drawn, and the box shaken after each drawing; thus, the probability of allocation to either study condition was always 50%.

All participants were given a username and password-protected access to their respective websites. Data were stored on a secure server and password-protected computer. Participants assigned to the control condition were sent a link to an educational resource relating to cannabis use. Upon clicking this link, entry to this website occurred via checking an informed consent box and completion of the baseline assessment questionnaire. Participants assigned to the experimental condition were similarly sent a link to the intervention website, which contained the baseline assessment questions prior to accessing the remainder of the website. After this point, routine study procedures were fully automated. No further contact was made with participants for 6 weeks, at which point they were contacted by an automatically generated email that requested completion of follow-up data by returning to the website. Participants who did not respond were sent up to 3 reminder emails on a weekly basis. A researcher telephoned Australian participants who did not respond to these email requests and asked them to log in to the website and complete the assessment.

**Figure 1:**
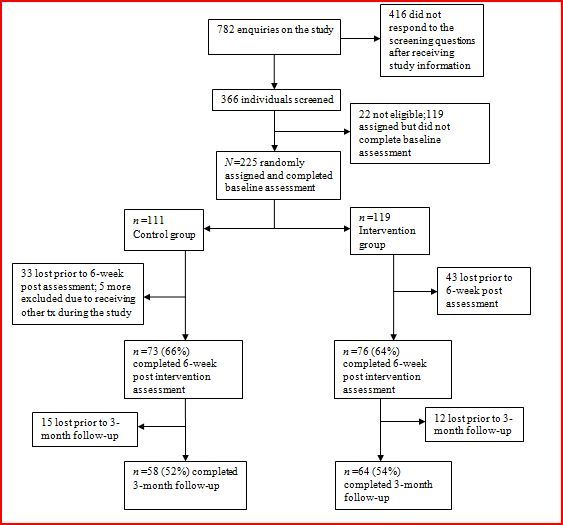
CONSORT diagram of participant flow.

Three months post randomization, participants were contacted in the same manner as described for the 6-week follow-up. Participants completing each research assessment were given a gift voucher worth $30 AUD (Australian participants) or $30 AUD via PayPal (participants from other countries). Those assigned to the control condition were sent a link to the intervention website at the conclusion of the study. Figure 1 shows a CONSORT [19] diagram describing participant flow.

### Interventions

**Figure 2:**
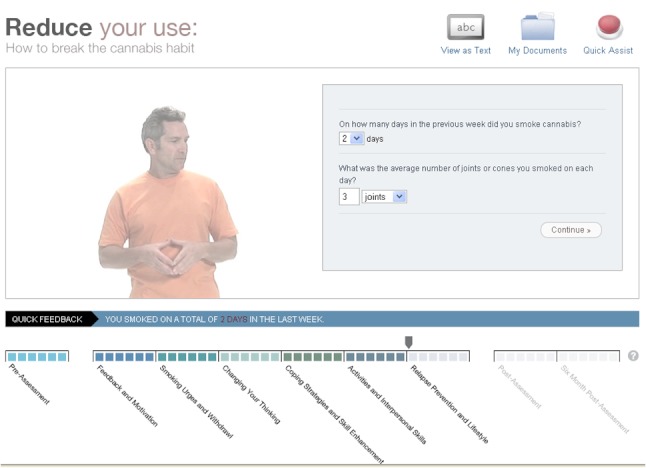
Screenshot of the intervention.

The intervention website, *Reduce Your Use: How to Break the Cannabis Habit* (Figure 2), is a newly developed intervention, largely based on a face-to-face brief treatment previously found to be effective for problematic cannabis use [17]. The face-to-face treatment was informed by the principles of CBT and motivational interviewing (MI) and was specifically based on previous cognitive-behavioral interventions with known efficacy in managing substance use [20,21]. The web adaptation was also informed by other web-based interventions targeting substance use that used automated feedback [22]. The website contains 6 core modules, which are undertaken sequentially at intervals chosen by the participant. These are briefly summarized in Appendix 1. Feedback on the participant’s progress is available throughout the sequence via graphing of cannabis use through the program and detailed feedback on changes in use and related factors such as attitude toward cannabis, goal setting, and weekly expenditure on cannabis. The website also features a personalized folder for the participant, blogs from former cannabis users, quick assist links, and weekly automatically generated encouragement emails. Individuals using the website have the option of reading its text or watching a video of an actor speaking the text.

The control condition website contains information about cannabis and consists of 6 sections, with content as follows: (1) What is cannabis? (2) Cannabis potency, (3) Cannabis and the law, (4) Cannabis in the workplace, (5) Cannabis and aggression, and (6) Cannabis and driving. The information provided does not contain any content aimed at building skills or changing motivation or other aspects of thinking about cannabis, nor in supporting actual behavior change attempts. Participants did not need to read the sections in sequential order, and we did not monitor the number of sections each participant read.

### Outcome Evaluation and Measures

Trial outcomes were assessed 6 weeks and 3 months post randomization. If participants completed 1 module per week as recommended, the 6-week follow-up approximates a short-term posttreatment assessment. Participants may not have completed all modules or completed them more quickly than in 6 weeks. The reference period for all measures at all assessment points was the past month. There was no blinding of participants, who were thus aware that they had an equal probability of being assigned to the intervention or the educational website. Outcome data collection was automated, negating the need to blind researchers.

Days of cannabis use over the past month and past-month quantity of cannabis use were primary outcomes measures. These were assessed using the Timeline Follow-Back method (TFLB) [23], adapted to measure standard cannabis units (SCUs), where a regular-sized joint or 3 cones equals 1 SCU [24]. As well as the extensively validated frequency measure, quantity estimates from the TFLB have been found to be reliable [25]. Although the TFLB is a somewhat complex measure, previous research supports the validity of its use over the Internet [26]. Other outcomes included past-month abstinence, number and severity of past-month cannabis dependence symptoms, and past-month number of cannabis abuse symptoms. Cannabis dependence severity was measured using the Severity of Dependence Scale (SDS) [27]. Number of cannabis dependence and abuse symptoms were assessed using the GAIN-I (Global Appraisal of Individual Needs - Initial) [28]. Participants also indicated the age at which they initiated cannabis use and provided basic demographic information.

Participants in the intervention group completed questions relating to their compliance and engagement with the program during the 6-week assessment. These included 1-4 ratings for the questions: “How closely did you follow the content of each module?” (1 = not closely at all, 4 = very closely), and “To what extent did you carry out the website’s skill-building tasks?” (1 = did not do any of the tasks, 4 = did all of the tasks). Participants also were asked how many of the 6 modules they completed. In addition to providing information relating to compliance, participants in the intervention group rated their satisfaction with each module out of 10 and also completed the Client Satisfaction Questionnaire (CSQ-7) [29]. This included 7 items relating to satisfaction with the program, eg, “How would you rate the quality of the service you have received? To what extent has our program met your needs?” Items were rated on a 4-point scale, with higher ratings indicating higher satisfaction. Each measure in the assessment was placed on a single screen; thus, the number of items per page varied, depending upon the measure.

### Data Analysis

Statistical significance was defined as a two-tailed *P* value below .05. Complier average causal effect (CACE) analysis, performed using Mplus software [30], was employed for continuous outcome measures. CACE contrasts study outcomes for treatment group participants who are classed as compliers relative to participants in the control group who would have complied had they been assigned to the treatment group (see articles by Connell [31] and Jo et al [32] for a more detailed explanation of how “would be” compliance in the control condition is operationalized as a latent variable and for further information on CACE procedures). Standard assumptions were met with regard to the use of CACE analysis in the current study.

For our analyses, we defined a noncomplier as a participant who completed only 1 module or less and/or indicated that they did not follow the intervention at all closely, and/or failed to complete any of the skill-building exercises, and/or failed to complete at least some of the 6-week postintervention assessment or at least some the 3-month follow-up assessment. The rationale behind selecting a cut-off of 1 module was that participants who completed more than 1 module returned to the program at least once.

CACE has been recommended for use in RCTs, where noncompliance and attrition are extremely common [18,31]. It has also been argued that the notoriously high rate of attrition associated with web-based intervention studies deems it necessary to employ analyses that estimate the efficacy of the intervention on individuals who actually use it [18]. Therefore, CACE was considered appropriate as the primary analysis in the current study. This decision was not made a priori; rather, it was made due to the observed level of missing data. A missing values analysis showed that data were missing completely at random (Little’s MCAR test χ^2^ = 194.21, *P*=.66). The CACE analysis addressed missing data by imputing missing values on continuous variables. The procedure used for imputation was PASW 17’s Expectation Maximization (EM) imputation procedure. This is a maximum likelihood approach that uses an iterative algorithm to estimate the parameters of the complete dataset [33].

In light of attrition from the study, conducting a traditional intention-to-treat analysis (ITT) with postintervention and follow-up data from all cases was not possible. Instead, EM without CACE is also reported as the primary ITT analysis. This analysis employed between-groups repeated measures ANOVAs.

Listwise deletion (excluding participants who did not complete the relevant assessment) and last-score-carried forward analyses were also conducted in order to test outcomes as comprehensively as was feasible. These analyses were conducted through between-groups repeated measures ANOVAs. To avoid overcomplicating the results, outcomes of these two analyses are not reported in detail. Rather, a brief comparison of these findings and our primary analyses are presented in the Results section. While last-score-carried-forward analyses are often employed as a primary analysis in RCTs, we decided against this in the current study in light of several recent studies indicating that the technique can be vulnerable to bias where there are large amounts of missing data and thus should be avoided [34-36].

All between-groups analyses were conducted with the outcome variables adjusted for the baseline score. Other baseline variables previously found to be associated with continued cannabis use (age of cannabis use initiation, gender, and age) [37] were also entered into analyses as covariates.

Group differences in past-month abstinence were assessed in logistic regression models, performed on PASW 17 [38]. Additional analyses involved examining bivariate correlations among adherence/satisfaction and outcome variables. These also were conducted on PASW 17. Imputation was not used for these data.

### Reporting

The research is reported in accordance with the E-CONSORT checklist [39] (see Appendix 2).

## Results

Demographic characteristics and assessment data at baseline are presented in Table 1. Randomization was successful, with groups not differing significantly on any baseline variable.

**Table 1:** Baseline means (SD) or percentage scores on participant demographics and cannabis-related variables (N=225).

	Intervention (n=119)	Control (n=111)	*df*	*t*	χ^2^	*P*
Age	31.88 (9.85)	30.18 (9.62)	224	1.33		.19
Gender (male)	59.7%	63.2%	1		0.30	.58
Age/initiation	16.31 (3.71)	16.22 (3.20)	224	0.20		.84
Frequency (days past month)	21.33 (8.24)	20.76 (8.68)	224	0.49		.62
Quantity (SCUs^a^ past month)	79.28 (72.68)	70.66 (60.96)	224	0.99		.32
SDS^b^	13.97 (3.61)	13.78 (3.61)	224	0.37		.71
GAIN-dependence	4.47 (1.57)	4.40 (1.65)	224	0.36		.71
GAIN-abuse	2.61 (1.41)	2.43 (1.41)	224	0.96		.34

^a^ SCUs = standard cannabis units.

^b^ SDS = Severity of Dependence Scale.

Sixty-six percent (149 of 225) of participants completed the 6-week postintervention assessment, while 51% (122 of 225) completed the 3-month follow-up assessment. Five control group participants were excluded from the study because they reported receiving other professional treatment during the course of the intervention. No participants in the experimental group reported receiving additional treatment. Completion rates did not differ significantly between groups for either assessment (*P*s>.10). All but 2 participants who completed the 3-month assessment also completed the 6-week assessment (ie, only 2 participants did not complete the postassessment but completed the follow-up assessment). Table 2 shows outcomes for cannabis-related variables at 6 weeks and 3 months with EM imputation. Participants in both conditions reported significant change on all outcome variables after 6 weeks and maintained significant change after 3 months.

**Table 2:** Cannabis-related variables across assessments (N=225; imputation is not used for the Abstinence variable).

Variable	Intervention	Control	Intervention	Control	Intervention	Control
	Baseline	Baseline	6 weeks	6 weeks	3 months	3 months
	(n=111)	(n=119)	(n=76)	(n=73)	(n=64)	(n=58)
Frequency (days past month)	21.33 (8.24)	20.76 (8.68)	12.90 (8.47)^a^	14.87 (8.88)^b^	12.05 (8.99)^a^	14.11 (8.79)^b^
Quantity (SCUs^c^ past month)	79.28 (72.68)	70.66 (60.96)	39.78 (44.97)^a^	46.16 (49.31)^b^	36.65 (44.85)^a^	39.25 (39.21)^b^
SDS^d^	8.97 (3.61)	8.78 (3.61)	7.31 (3.22)^a^	7.44 (3.56)^b^	5.70 (3.35)^a^	6.82 (3.31)^b^
GAIN-dependence	4.47 (1.57)	4.40 (1.65)	3.09 (1.69)^a^	3.21 (1.60)^b^	2.53 (1.67)^a^	3.10 (1.67)^b^
GAIN-abuse	2.61 (1.41)	2.43 (1.41)	1.60 (1.22)^a^	1.79 (1.37)^b^	1.24 (1.03)^a^	1.56 (1.24)^b^
Abstinence	N/A	N/A	9.3%	4.7%	12.4%	6.6%

^a^ Significantly different from intervention group baseline assessment (*P*<.001).

^b^ Significantly different from control group baseline assessment (*P*<.001).

^c^ SCUs = standard cannabis units.

^d^ SDS = Severity of Dependence Scale.

### CACE Analyses With EM

A series of group comparisons employing CACE analyses, controlling for the previously noted covariates, are reported in Table 3. For the 6-week postassessment, there were 61 compliers and 68 noncompliers in the treatment group; for the 3-month follow-up, there were 53 compliers and 76 noncompliers in the treatment group. Hypothesis 1 predicted that at 6-week and 3-month follow-up assessments, participants in the intervention group would report significantly lower cannabis use frequency than would participants in the control group. This hypothesis was supported, with between-group differences significantly favoring the experimental group at both postintervention and follow-up.

Hypothesis 2 predicted that at the 6-week and 3-month follow-up assessments, participants in the intervention group would report a significantly lower quantity of cannabis use than would participants in the control group. The hypothesis was partially supported, with results showing significantly lower numbers of SCUs in the intervention group relative to the control group at the 6-week postassessment. This effect was, however, reduced somewhat by the 3-month follow-up, such that it no longer reached statistical significance.

Hypothesis 3 predicted that at 6-week and 3-month follow-up assessments, participants in the intervention group would report significantly lower levels of cannabis dependence than would participants in the control group. Group differences were not apparent after 6 weeks on either measure of cannabis dependence; however, significant differences did emerge for both measures at the 3-month follow-up, providing support for a slight delay in effects on cannabis dependence.

Hypothesis 4 predicted that at 6-week and 3-month follow-up assessments, participants in the intervention group would report significantly fewer symptoms of cannabis abuse than would participants in the control group. This hypothesis was supported, with between-group differences on the GAIN abuse measure significantly favoring the experimental group at both assessment points.

**Table 3:** CACE analyses of cannabis outcome measures at 6-week and 3-month assessments (N=225; intervention group coded as 1, control group coded as 2).

Variable		*B*	*SE*	*P*	*d* ^a^
**6-weeks post**					
	Smoking days	3.82	1.67	.02	0.38
	SCUs	24.86	9.78	.01	0.34
	SDS	0.43	0.73	.56	0.08
	GAIN-dependence	0.12	0.42	.78	0.04
	GAIN-abuse	0.60	0.30	.047	0.27
**3-month follow-up**					
	Smoking days	5.37	2.36	.02	0.31
	SCUs^b^	11.84	8.45	.16	0.19
	SDS^c^	2.37	0.84	.01	0.38
	GAIN-dependence	0.99	0.50	.047	0.27
	GAIN-abuse	1.05	0.40	.01	0.35

^a^ *d=* Cohen’s *d.*

^b^ SCUs = standard cannabis units.

^c^ SDS = severity of dependence scale.

### ITT Analyses With EM

An ITT analysis employing between-groups repeated measures ANOVAS and using EM imputation is reported in Table 4. All significant outcomes found using CACE were replicated in these analyses, with the exception of the analysis examining cannabis abuse at the 6-week assessment, which marginally missed statistical significance (*P*=.05).

**Table 4:** ITT analyses with EM showing group x time interactions on cannabis outcome measures at 6-week and 3-month assessments (N=225; intervention group coded as 1, control group coded as 2).

Variable		*F*	*df*	*P*	*d* ^a^
**6-weeks post**					
	Smoking days	5.12	220	.02	0.30
	SCUs^b^	6.31	220	.01	0.34
	SDS^c^	0.50	220	.49	0.10
	GAIN-dependence	0.43	220	.51	0.09
	GAIN-abuse	3.82	220	.05	0.26
**3-month follow-up**					
	Smoking days	5.88	220	.02	0.33
	SCUs	3.49	220	.06	0.25
	SDS	6.07	220	.01	0.33
	GAIN-dependence	5.18	220	.02	0.31
	GAIN-abuse	6.32	220	.01	0.34

^a^ *d=* Cohen’s *d*.

^b^SCUs = standard cannabis units.

^c^SDS = severity of dependence scale.

### Additional Analyses

Analyses using listwise deletion and last-score-carried-forward imputation were conducted as comparisons with the primary analyses. Several significant findings obtained in the primary analyses were replicated. The listwise deletion analyses found significant group x time interactions favoring the experimental group on smoking days at 6 weeks (*F* (144) = 4.45, *P*=.04), SCUs at 6 weeks (*F* (142) = 5.08, *P*=.03), and SDS at 3 months (*F* (117) = 4.56, *P*=.04). Other outcomes found to be significant in the primary analyses nonsignificantly favored the experimental group, with *P* values ranging from .05 to .17. Last-score-carried-forward analyses showed significantly lower SDS scores in the experimental group at 3 months (*F* (220) = 5.62, *P*=.02) and significantly lower cannabis abuse scores in the experimental group at 3 months (*F* (220) = 5.32, *P*=.02). Again, other outcomes found to be significant in the primary analyses showed nonsignificantly better results for the experimental group, with *P* values ranging from .08 to .15.

### Past-Month Abstinence

The final study hypothesis predicted that at 6-week and 3-month follow-up assessments, participants in the intervention group would report significantly higher rates of past-month abstinence than those in the control group. At the 6-week post intervention assessment, the intervention group had a higher rate of abstinence (9.3%; 7/76) than did the control group (4.7%; 3/73), though the numbers were small and the difference not statistically significant (OR 2.53, *P*=.10). Likewise, at the 3-month follow-up, past-month abstinence was higher in the intervention group (12.4%; 8/64) compared with the control group (6.6%; 4 out of 58), with the difference missing the conventional threshold of statistical significance (OR 2.50, *P*=.06).

### Process Analysis

Participants in the intervention group completed an average of 3.5 of the 6 modules. The percentage of participants who completed only the first module or less was 17.3%. The percentages of participants ceasing treatment after completing Modules 2-6 were 27.2%, 11.1%, 6.2%, 9.9%, and 28.4%, respectively. While we have reported participants’ self-reports of number of modules completed, these were closely corroborated by the program’s documentation of participant logins (*r*=.87). Number of modules completed was only significantly associated with one variable, reduction in past-month cannabis smoking days at both 6 weeks (*r*=.25, *P*=.04) and 3 months (*r*=.32, *P*=.01). Reported satisfaction with the program was generally high, with the mean score on the CSQ-7 being 3.41 (SD 0.64) out of a possible 4. Perhaps because of this, CSQ-7 scores were not significantly associated with any outcome variable (all *P*s>.07). Out of a possible 10, modules 1-6 received mean (SD) satisfaction ratings of 7.10 (2.21), 6.95 (2.28), 7.33 (2.47), 7.00 (2.47), 7.38 (2.61), and 7.85 (2.61), respectively.

## Discussion

This study evaluated the effectiveness of the *Reduce Your Use: How to Break the Cannabis Habit* program in a fully automated environment*.* We tested the hypotheses that at 6-week and 3-month follow-up assessments, relative to a an information-only control group, participants who were randomized to the intervention would report lower frequency of cannabis use (H1), lower quantity of cannabis use (H2), lower levels of cannabis dependence (H3), fewer symptoms of cannabis abuse (H4), and higher rates of past-month abstinence (H5).

The first hypothesis was supported. Primary analyses conducted in relation to cannabis use frequency revealed a significantly lower number of past-month smoking days in the intervention group, after controlling for pre-intervention smoking days. Reductions in smoking days were substantial, with the intervention group reducing from 21.33 smoking days per month at baseline to 12.05 days at 3-month follow-up. This equates to a 43.5% reduction in smoking days per month for the treatment group. The control group reduced their smoking days from 20.76 at baseline to 14.11 at follow-up, representing a 32.0% reduction in smoking days. The outcome for the intervention group on cannabis use frequency is comparable to outcomes of several face-to-face treatments for cannabis at similar time points [40-42].

Hypothesis 2 was supported in part. Results showed significantly lower numbers of SCUs in the intervention group relative to the control group at 6 weeks in each analysis. However, by 3 months, this effect had reduced to a nonsignificant level. The intervention group reduced their past-month number of SCUs from 79.28 at baseline to 36.65 at 3 months, which equates to a 53.8% reduction in SCUs per month. The control group reduced from 70.66 at baseline to 39.25 at follow-up, which represents a 44.2% reduction in past-month SCUs.

Partial support was found for Hypothesis 3 regarding cannabis dependence. Interestingly, the effect on cannabis dependence symptoms appeared to be delayed, with both measures of cannabis dependence showing a nonsignificant effect at the 6-week assessment and both showing a significant effect in favor of the intervention group at 3 months in each analysis. This finding may suggest that the intervention has some effects that endure or strengthen over time [43]. It is also consistent with findings in face-to-face interventions, where concern over use, as measured in the SDS, has a long lag as participants take the time to become more established in, and less concerned about, their changed pattern of use [14]. However, a longer follow-up period would be required to investigate these possibilities.

The fourth hypothesis, relating to cannabis abuse, was partially supported, with the CACE analysis finding significant group differences at each assessment point, but the ITT analysis not reaching statistical significance at the 6-week analysis (*P*=.05). The trend in the expected direction suggests insufficient power may have affected the outcome of this analysis.

The final hypothesis, regarding abstinence, was not supported. Neither the post nor the follow-up assessment indicated significantly higher levels of abstinence among the intervention group relative to the control group. Rates of abstinence were lower than in several other intervention studies for cannabis [17,44,45], including the face-to-face intervention upon which the program is based [17], which achieved 20.8% abstinence in the intervention group at 3-month follow-up. This may in part reflect the intervention’s focus on quitting *or* reducing cannabis use. It does, however, suggest that there is significant scope for improving online cannabis treatments to build rates of abstinence where that is the desired goal.

Findings of this study add to those of previous studies assessing computer-delivered treatments for cannabis, which have shown that such treatments are valuable as an adjunct to in-person therapy [13,14]. This study further demonstrates that fully self-guided treatment delivered online can assist individuals to reduce their cannabis use.

### Strengths and Limitations

This study has several important strengths. First, the intervention was designed to be fully self-guided, thus requiring minimal therapist input beyond the initial design of the program. Second, the program was able to reach a wide audience, both nationally and internationally. Third, continued operation and dissemination of the program can be achieved at low cost. Finally, this is an effectiveness study, designed to precisely estimate effects that may be obtained in real-world use outside of a research environment. These points engender confidence that the program will have positive effects as a free and publicly available cannabis treatment option. While rates of abstinence were lower than those achieved by highly trained clinicians using manualized interventions in traditional treatment settings, the significant reductions in the levels of cannabis use and related harms suggest that this web-based intervention offers great promise for reducing the public health burden of cannabis use.

There are also some important limitations to note in relation to the current study. First, the level of participant attrition was quite high with regard to completion of the treatment program as well as completion of assessments; however, high levels of attrition are the rule rather than the exception in web-based treatment studies. For example, online treatment programs have reported program completion rates as low as 0.5% and drop-out rates as high as 65% [18]. Finding ways of decreasing participant attrition in web-based studies should be an imperative for future related studies.

A second limitation is that the long-term effectiveness of the treatment program cannot be known with a 3-month follow-up period—future studies should contain provisions for longer durations of follow-up assessment with and without booster sessions. Similarly, findings on the effectiveness of this intervention are entirely restricted to the self-guided study context. It is possible that facilitation of uptake of such interventions may enhance their effectiveness. An additional limitation is that the study relied solely on self-report data for all outcome measures. With regard to our cannabis use outcome measures, however, there was little that could be done to rectify this as urinalysis was not possible in light of participation being open to individuals around Australia and elsewhere. Saliva analysis was not feasible due to funding and time constraints and is of questionable validity in the context of the current study. Finally, tobacco smoking was not taken into consideration as a factor that could influence success in reducing cannabis use and maintaining reductions. Future research would benefit from gauging the level of influence tobacco use exerts on cannabis use outcomes.

### Implications and Future Directions

Findings of the current study provide support for further investigation of web-based treatment for cannabis use and related problems. Should these evaluations yield positive outcomes, the availability of web-based treatment for cannabis use could lead to valuable and tangible developments in service delivery and treatment uptake. These could include an ease on the public health burden of cannabis use disorders, reductions in treatment waitlists, and increased uptake of treatment due to the high acceptability of online treatments to many users.

While there are many important areas of investigation for future studies, treatment adherence and retention are worthy of prominent consideration. In the current study, the average number of modules completed was 3.5 out of 6 and the retention rate at follow-up was 53%. This is lower than the average number completed in the face-to-face study upon which the current study is based (4.2 out of 6 modules), as well as the follow-up retention rate of 74%. There was some evidence of a relationship between adherence and treatment outcome, where number of modules completed did correlate with reduced frequency of use. Noncompletion of treatment in web-based studies is extremely common, and recent work suggests that additions to web-based intervention studies, such as brief weekly telephone check-ins [46] and use of incentives [47], can increase engagement with treatment as well as assessment completion rates. Future research on web-based studies addressing cannabis use could test whether additions such as these help to promote engagement and to reduce drop-out. Such methods may also lead to enhanced treatment outcomes. Increasing treatment satisfaction is another possible means of increasing treatment adherence. In the current study, satisfaction with the intervention was generally high as measured by the CSQ-7 and participant ratings of module quality. All modules received a rating of at least 7/10, with the exception of Module 2, which addressed coping with cravings and withdrawal symptoms. This is another area in which future web-based interventions could be improved. For example, the module included little information on sleep problems, which one study recently found was the primary symptom associated with cannabis withdrawal [48].

There are few similar web-based interventions targeting substance use. In the alcohol field, *Down Your Drink* targeted anyone considering their drinking and did not require a decision to change to have been made prior to participation in the trial [49]. In contrast, an intervention known as *MinderDrinken* recruited help-seekers who had already made a decision to change [50]. Findings from the evaluation studies were positive for the latter study and negative for the former, with similarities in the content of the interventions themselves. Thus, it should be taken into consideration that findings of the current study may differ if the intervention program were to be used by non-help-seeking cannabis users. Future studies could examine whether online cannabis intervention programs have a significant impact on non-treatment-seekers.

Other important issues for future research include investigations of longer term effects of treatment, examining the effects of adaptations to suit varying demographics and cultures, and exploring the feasibility and impact of combining web-based treatment with face-to-face therapy.

### Conclusions

Web-based treatments for substance use disorders are becoming increasingly available; however, up to this point, no completely self-guided web-based cannabis treatment has been tested in a randomized controlled trial. Outcomes of the current study suggest that *Reduce Your Use* holds promise in assisting individuals who wish to quit or reduce their cannabis use and also point to possible means of improving outcomes of web-based interventions for cannabis and other substance use disorders.

## Multimedia Appendix 1

The structure of Reduce Your Use: How to Break the Cannabis Habit.

## Multimedia Appendix 2

CONSORT EHEALTH Checklist V1.6.2 [51].

## Abbreviations

CACE: complier average causal effect
CBT: cognitive behavioral therapy
CSQ: Client Satisfaction Questionnaire
EM: expectation maximization
GAIN: Global Appraisal of Individual Needs
ITT: intention-to-treat analysis
MI: motivational interviewing
NCPIC: National Cannabis Prevention and Information Centre
RCT: randomized controlled trial
SCU: standard cannabis units
SDS: Severity of Dependence Scale
TFBM: timeline follow-back method
UNSW: University of New South Wales
