# Supplementary material for: Effectiveness of a Self-Guided Web-Based Cannabis Treatment Program: Randomized Controlled Trial
Source: J Med Internet Res. 2013 Feb 15;15(2):e26. doi: 10.2196/jmir.2256 (PMC3636012; doi:10.2196/jmir.2256)
Supplement: Supplementary file 1 [file jmir_v15i2e26_app1.pdf]

Appendix 1. The structure of *Reduce Your Use: How to Break the Cannabis Habit*

Module 1: Feedback and Building Motivation

The goal of Module 1 is to assist the participant in preparing for change. Beyond the trial assessment data, the participant completes additional self-assessment questionnaires about current cannabis use and change. They then receive personalized feedback relating to these issues and set a goal and date for reducing or quitting cannabis. Self-monitoring is also encouraged through the provision of an electronic cannabis smoking diary.

Module 2: Managing Smoking Urges and Withdrawal

The main goals of Module 2 are to build the participant's understanding of cannabis withdrawal symptoms and cannabis cravings and to prepare for ways of coping with or avoiding cravings. The module also provides a self-test of the participant's confidence that they can resist using cannabis in a variety of situations.

Module 3: Changing Your Thinking

This provides a discussion of automatic thoughts that can undermine attempts to break a cannabis use pattern. Examples of such thoughts are provided. To assist the participant in effectively coping with automatic thoughts that can lead to cannabis use, a skill-building activity involving challenges to automatic thoughts is included. As an optional extra, the participant can complete an exercise aimed at identifying automatic thoughts about cannabis.

Module 4: Coping Strategies and Skill Enhancement

This provides an overview of "slippery slope" decisions, small steps in chains of events that can undermine change. The participant is given the opportunity to practice avoiding slippery slope decisions during a skill-building task. For wider personal skill development, the module also contains three optional extras: coping skills training, improving sleep, and relaxation training.

Module 5: Activities and Interpersonal Skills

Here the participant is given an opportunity to review and consolidate what they have learned so far. If the participant has experienced any problems that can be addressed by previous aspects of the website, we encourage a review of those parts of the website at this point. Another skill-building activity follows. This involves the participant thinking of a skill or an activity that they are only comfortable performing when stoned and then trying to perform the activity not stoned, followed by evaluating the outcome. Two further optional extras are offered here: assertiveness skills and emotion management.

Module 6: Relapse Prevention and Lifestyle Changes

The final module focuses on making change permanent. Intentional and unintentional lapses are discussed. The participant is given methods that may be useful in preventing and responding to both kinds of lapses.
