# Supplementary material for: Effectiveness of a Self-Guided Web-Based Cannabis Treatment Program: Randomized Controlled Trial
Source: J Med Internet Res. 2013 Feb 15;15(2):e26. doi: 10.2196/jmir.2256 (PMC3636012; doi:10.2196/jmir.2256)
Supplement: Supplementary file 2 [file jmir_v15i2e26_app2.pdf]

CONSORT-EHEALTH Checklist V1.6 Report	Manuscript Number	2256
Date completed	8/25/2011 14:50:29	
by	Sally Rooke	
Randomized Controlled Trial of Reduce Your Use: a Self-Guided Web-Based Cannabis Treatment Program		
TITLE		
**1a-i) Identify the mode of delivery in the title**		
Title: ".. a Self-Guided Web-Based Cannabis Treatment Program"		
**1a-ii) Non-web-based components or important co-interventions in title**		
Not applicable: All components of the intervention were web-based.		
**1a-iii) Primary condition or target group in the title**		
Title: "...Cannabis Treatment Program"		
ABSTRACT		
**1b-i) Key features/functionalities/components of the intervention and comparator in the METHODS section of the ABSTRACT**		
"The current study aimed to test the effectiveness of Reduce Your Use, a fully self-guided web-based treatment program for cannabis use disorder consisting of six modules based on cognitive, motivational, and behavioral principles." "...were randomly assigned to receive (a) the web-based intervention, or (b) a control condition consisting of six modules of web-based educational information on cannabis."		
**1b-ii) Level of human involvement in the METHODS section of the ABSTRACT**		
The term "fully self-guided" is used in the Objectives section of the Abstract.		
**1b-iii) Open vs. closed, web-based (self-assessment) vs. face-to-face assessments in the METHODS section of the ABSTRACT**		
"..were recruited using both online and offline advertising methods"<br><br>"..Reduce Your Use, a fully self-guided web-based treatment program.."<br><br>"Assessments of cannabis use, dependence symptoms, and abuse symptoms were conducted through online questionnaires.."		
**1b-iv) RESULTS section in abstract must contain use data**		
"Two thirds (149) of the participants completed the six-week post-intervention assessment, while 122 (54%) completed the three-month follow-up assessment. Participants in the intervention group completed an average of 3.5 of the 6 modules."<br><br>All outcome variables are noted in the Abstract (past month frequency/quantity, abstinence, number of abuse symptoms, number of dependence symptoms, and severity of dependence symptoms.)		
**1b-v) CONCLUSIONS/DISCUSSION in abstract for negative trials**		
Not applicable. Findings were primarily as hypothesised.		
INTRODUCTION		
**2a-i) Problem and the type of system/solution**		
"..many cannabis users are employed and unable to attend face-to-face sessions during working hours. Second, residents of remote areas or localities poorly serviced by public transport have difficulty traveling to regular sessions. Third, face-to-face therapy is economically burdensome and provision services frequently cannot meet demand [6]. Finally, many people hesitate to seek treatment due to concerns about confidentiality and being stigmatized [7]. These issues underscore the vital need for evidence-based treatments that are highly accessible, financially efficient, and have a high level of acceptability to consumers." "In response to the absence of evidence-based fully self-guided online treatments for cannabis use, the authors developed Reduce Your Use: How to Break the Cannabis Habit."		
**2a-ii) Scientific background, rationale: What is known about the (type of) system**		

"Internet-delivered treatments may assist in resolving these issues, offering several advantages, including bridging the gap between supply and demand for alcohol and drug therapists, being potentially more cost effective than face-to-face treatment, and having the ability to be accessed at most times and in most locations. Increased privacy largely addresses the issue of stigmatization. Additionally, where treatment is automated, it is consistently delivered in its intended manner [8].

Several computer programs and web-based interventions for substance use have recently been developed and tested for their efficacy. The treatments consist of components such as cognitive behavioral therapy (CBT) [9], chat forums [10], and normative feedback on substance use [11]. A recent meta-analysis of the efficacy of computer-delivered treatments for tobacco and alcohol use found that, overall, the treatments had a significant effect [12]. A non-randomized study by Budney et al. involving 38 participants found that a computerized intervention program with therapist support yielded similar reductions in cannabis use to a therapist delivered intervention [13]. Tossmann et al. tested the effects of a therapist assisted online treatment program for cannabis use in a randomized trial with high levels of attrition, finding significant effects on cannabis use reductions [14]. Sinadinovic et al. found some evidence that an online brief intervention program was superior to assessment-only in assisting illicit drug users to reduce their substance use [15]. No previous study, however, has empirically tested the efficacy of a fully self-guided web-based treatment for cannabis use and related problems."

Comparator: information only as attention control -

"..information provided does not contain any content aimed at building skills or changing motivation or other aspects of thinking about cannabis, nor in supporting actual behavior change attempts."

METHODS

3a) CONSORT

"We hypothesized that at six-week and three-month follow-up assessments, relative to an information-only control group, individuals who were randomized to the intervention would report lower frequency of cannabis use (H1), lower quantity of cannabis use (H2), lower levels of cannabis dependence (H3), and fewer symptoms of cannabis abuse (H4). We further hypothesized that the intervention group would report higher rates of past-month abstinence at both follow-up points (H5)."

3b-i) Bug fixes, Downtimes, Content Changes

Not relevant. While several bug fixes occurred prior to participant recruitment, they did not occur after recruitment commenced.

4a-i) Computer / Internet literacy

"Study participants were adults who were at least 18 years old, and were English and computer literate.."

4a-ii) Open vs. closed, web-based vs. face-to-face assessments:

"Recruitment advertisements seeking individuals who wished to reduce or quit their cannabis use via an online program were placed on the National Cannabis Prevention and Information Centre (NCPIC) website, online forums, Google, university bulletin boards, in newspapers, and at community health centers."

"Interested individuals contacted the research team via email and were sent screening and study information materials by return email."

"After responding to the screening questions...participants were given a username and password protected access to their respective websites."

"...entry to this website occurred via checking an informed consent box and completion of the baseline assessment questionnaire...

"After this point, routine study procedures were fully automated. No further contact was made with participants for six weeks, at which point they were contacted by an automatically generated email that requested completion of follow-up data by returning to the website."

4a-iii) Information giving during recruitment

"Recruitment advertisements seeking individuals who wished to reduce or quit their cannabis use via an online program were placed on the National Cannabis Prevention and Information Centre (NCPIC) website, online forums, Google, university bulletin boards, in newspapers, and at community health centers. NCPIC and UNSW affiliations were displayed on all advertisements. Interested individuals contacted the research team via email and were sent screening and study information materials by return email. Inclusion/exclusion criteria (aside from being 18 or older) were not stated on the advertisement, nor specifically noted during participant screening, in order to prevent individuals from providing false information in order to be eligible for the study. Compensation for completing assessments was not noted in the study advertisement, but was noted in the participant information sheet, which participants received after contacting us to indicate their interest in the study.

Participants were informed that they would be randomly assigned to receive six modules of CBT or six modules of educational information. "

4b-i) Report if outcomes were (self-)assessed through online questionnaires

"Assessments of cannabis use, dependence symptoms, and abuse symptoms were conducted through online questionnaires at baseline, and at six-week and three-month follow-ups."		
**4b-ii) Report how institutional affiliations are displayed**		
"NCPIC and UNSW affiliations were displayed on all advertisements."		
**5-i) Mention names, credential, affiliations of the developers, sponsors, and owners**		
"In response to the absence of evidence-based fully self-guided online treatments for cannabis use, the authors developed Reduce Your Use: How to Break the Cannabis Habit."		
**5-ii) Describe the history/development process**		
The program was focus tested on 10 cannabis users during the development phase. In-house testing was conducted for approximately one month following the program's development, then 20 cannabis users tested the program. Recruitment for the study commenced after this.		
**5-iii) Revisions and updating**		
This is the first version of the program. No major changes to the program were made over the course of the study, and currently there are no plans to modify the program.		
**5-iv) Quality assurance methods**		
To increase accuracy of information, the study employed validated measures that were suitable for online implemenation. We also assured participants that all information obtained is confidential and stored on a secure server.		
**5-v) Ensure replicability by publishing the source code, and/or providing screenshots/screen-capture video, and/or providing flowcharts of the algorithms used**		
The website is noted in the paper and has been archived. Study protocol are also available from the first author on request.		
**5-vi) Digital preservation**		
"The website can be viewed at www.reduceyouruse.org.au"<br><br>The website is also archived.		
**5-vii) Access**		
"All participants had used cannabis at least once during the past month, and expressed a desire to stop or reduce their cannabis use."<br><br>"All participants were given a username and password protected access to their respective websites. Data were stored on a secure server and password protected computer." "Upon clicking this link, entry to this website occurred via checking an informed consent box and completion of the baseline assessment questionnaire."<br><br>"Participants completing each research assessment were given a gift voucher worth $30 AUD (Australian participants) or $30 AUD via PayPal (participants from other countries). Those assigned to the control condition were sent a link to the intervention website at the conclusion of the study."<br><br>"The website can be viewed at www.reduceyouruse.org.au"		
**5-viii) Mode of delivery, features/functionalities/components of the intervention and comparator, and the theoretical framework**		
"The intervention website, Reduce Your Use: How to Break the Cannabis Habit, is a newly developed intervention, largely based on a face-to-face brief treatment previously found to be effective for problematic cannabis use [17]. The face-to-face treatment was informed by the principles of CBT and motivational interviewing (MI), and was specifically based on previous cognitive-behavioral interventions with known efficacy in managing substance use [20, 21]. The web adaptation was also informed by other web-based interventions targeting substance use that used automated feedback [22]. The website contains six core modules, which are undertaken sequentially at intervals chosen by the participant. These are briefly summarized in Box 1. Feedback on the participant's progress is available throughout the sequence via graphing of cannabis use through the program and detailed feedback on changes in use and related factors such as attitude toward cannabis, goal setting, and weekly expenditure on cannabis. The website also features a personalized folder for the participant, blogs from former cannabis users, quick assist links, and weekly automatically generated encouragement emails. Individuals using the website have the option of reading its text or watching a video of an actor speaking the text. The control condition website contains information about cannabis, and is comprised of six sections, with content as follows: (1) What is cannabis? (2) Cannabis potency, (3) Cannabis and the law, (4) Cannabis in the workplace, (5) Cannabis and aggression, and (6) Cannabis and driving. The information provided does not contain any content aimed at building skills or changing motivation or other aspects of thinking about cannabis, nor in supporting actual behavior change attempts."		
**5-ix) Describe use parameters**		
"If participants completed one module per week as recommended, the six-week follow-up approximates a short term post-treatment assessment. Participants may not have completed all modules or completed them more quickly than in six weeks."		
**5-x) Clarify the level of human involvement**		

"This is an effectiveness study, designed to precisely estimate effects that may be obtained in real-world use outside of a research environment. These points engender confidence that the program will have positive effects as a free and publicly available cannabis treatment option." The only differences between the trial and outside use of the program are the initial screening email and the online assessments included in the trial.		
**5-xi) Report any prompts/reminders used**		
"The website also features .... automatically generated encouragement emails."<br><br>"No further contact was made with participants for six weeks, at which point they were contacted by an automatically generated email that requested completion of follow-up data by returning to the website. Participants who did not respond were sent up to three reminder emails on a weekly basis. A researcher telephoned Australian participants who did not respond to these email requests, and asked them to log in to the website and complete the assessment.<br><br>Three months post-randomization, participants were contacted in the same manner as described for the six-week follow-up."		
**5-xii) Describe any co-interventions (incl. training/support)**		
No co-interventions were included.		
**6a-i) Online questionnaires: describe if they were validated for online use and apply CHERRIES items to describe how the questionnaires were designed/deployed**		
CHERRIES items are used to the extent feasible in the Methods section.<br><br>"Although the TFLB is a somewhat complex measure, previous research supports the validity of its use over the Internet [26]."<br><br>Other questionnaires are very basic and the authors had no reason to believe they would not be valid for online use.		
**6a-ii) Describe whether and how "use" (including intensity of use/dosage) was defined/measured/monitored**		
This is described under Assessments in the Method section. The statisical analysis section also provides a definintion of program compliance.		
**6a-iii) Describe whether, how, and when qualitative feedback from participants was obtained**		
Participants in the intervention group were given the option of providing qualitative feedback at the end of each module. We have not reported on this feedback, but a summary is available from the first author upon request.		
**7a-i) Describe whether and how expected attrition was taken into account when calculating the sample size**		
"Our power calculation was based on a projected effect size of d = 0.45, as this was obtained for cannabis use frequency in the face-to-face treatment on which the intervention was based [17]. This required a total of 158 participants to achieve 80% power. However, given that web-based studies are prone to higher attrition rates than are face-to-face treatments [18], we recruited a larger number of participants (N = 225)."		
**7b) CONSORT**		
These did not apply to our study.		
**8a) CONSORT**		
"After responding to the screening questions, and prior to completing the baseline assessment, those eligible for participation were randomly assigned by the first author. Assignment occurred through the drawing of one of two tokens from a box. The tokens were two different colours, representing the two study conditions. The token was replaced each time it was drawn and the box shaken after each drawing; thus, the probability of allocation to either study condition was always 50%."		
**8b) CONSORT**		
"The probability of allocation to either study condition was always 50%."		
**9) CONSORT**		
"Assignment occurred through the drawing of one of two tokens from a box. The tokens were two different colours, representing the two study conditions. The token was replaced each time it was drawn and the box shaken after each drawing; thus, the probability of allocation to either study condition was always 50%."		
**10) CONSORT**		
The first author: "After responding to the screening questions, and prior to completing the baseline assessment, those eligible for participation were randomly assigned by the first author."		
**11a-i) Specify who was blinded, and who wasn't**		
Participants and researchers were not blinded.<br><br>"Outcome data collection was automated, negating the need to blind researchers."		
**11a-ii) Discuss e.g., whether participants knew which intervention was the "intervention of interest" and which one was the "comparator"**		

Participants were not explicitly told which online program was the one of interest and which the comparator. However, it is not unlikely that participants in the comparator group would have been aware that they had not received the intervention of interest as they were informed prior to participating that the alternative program would be available to them after three months.		
**11b) CONSORT**		
As with the intervention condition, the control condition contains six online modules; however, the information provided does not contain any content aimed at building skills or changing motivation or other aspects of thinking about cannabis, nor in supporting actual behavior change attempts."		
**12a) CONSORT**		
"Complier average causal effect (CACE) analysis, performed using Mplus software [30], was employed for continuous outcome measures."<br><br>"EM without CACE is also reported as the primary ITT analysis. This analysis employed between-groups repeated measures ANOVAs."		
**12a-i) Imputation techniques to deal with attrition / missing values**		
"...addressed missing data by imputing missing values on continuous variables. The procedure used for imputation was PASW 17's Expectation Maximization (EM) imputation procedure. This is a maximum likelihood approach that uses an iterative algorithm to estimate the parameters of the complete dataset [33]."		
**12b) CONSORT**		
CACE analyses include compliers as the subgroup: "CACE contrasts study outcomes for treatment group participants who are classed as compliers relative to participants in the control group who would have complied had they been assigned to the treatment group."<br><br>Adjustments were not made as groups did not differ significantly on any baseline variable.		
RESULTS		
**13a) CONSORT**		
"Sixty-six percent (149 of 225) of participants completed the six-week post intervention assessment, while 51% (122 of 225) completed the three-month follow-up assessment." The CONSORT diagram shows dropout by group.<br><br>"Participants [in the control group] did not need to read the sections in sequential order and we did not monitor the number of sections each participant read."<br><br><br>"Participants in the intervention group completed an average of 3.5 of the 6 modules. The percentage of participants who completed only the first module or less was 17.3%. The percentages of participants ceasing treatment after completing modules 2-6 were 27.2%, 11.1%, 6.2%, 9.9%, and 28.4%, respectively."		
**13b) CONSORT**		
The CONSORT diagram shows dropout by group. Additionally, "Five control group participants were excluded from the study because they reported receiving other professional treatment during the course of the intervention."		
**13b-i) Attrition diagram**		
Attrition is shown in the CONSORT diagram, and addressed throughoout the text. Number of modules participants completed is also addressed. We did not collect the information needed to plot usage over timepoints.		
**14a) CONSORT**		
"Participants were recruited between April 2010 and May 2011." Therefore, the last followups occurred in August 2011.		
**14a-i) Indicate if critical "secular events" fell into the study period**		
Critial secular events did not occur over the study period.		
**14b) CONSORT**		
The trial was not stopped early; itended when we recruited 225 participants. We considered this number to be sufficient in order for us to have adequate power.		
**15) CONSORT**		
Provided in Table 1.		
**15-i) Report demographics associated with digital divide issues**		
Age and gender are reported. Computer literacy was an inclusion criterion.		
**16-i) Report multiple "denominators" and provide definitions**		

Analyses for compliers and non-compliers are employed, as well as an ITT analyses. All primary analyses use the entire sample (this is specified in the tables containing the analyses).<br><br>The paper reports on number of compliers, dropout rate, and number of modules completed by participants. Correlations between number of modules completed and outcome variables are also examined.		
**16-ii) Primary analysis should be intent-to-treat**		
One of the primary analyses is an intention-to-treat. Secondary analyses include listwise deletion and intention-to-treat without data imputation.		
**17a) CONSORT**		
Tables provide results for each group along with effect sizes for between-groups differences.		
**17a-i) Presentation of process outcomes such as metrics of use and intensity of use**		
We collected data on number of modules completed and on other items relating to how closley content was followed etc; however, we did not collect information on session length.		
**17b) CONSORT**		
"At the six-week post intervention assessment, the intervention group had a higher rate of abstinence (9.3%; 7/76) than did the control group (4.7%; 3/73), though the numbers were small and the difference not statistically significant (OR = 2.53, P = .10). Likewise, at the three-month follow-up, past month abstinence was higher in the intervention group (12.4%; 8/64) compared with the control group (6.6%; 4 out of 58)."		
**18) CONSORT**		
Exploratory/adjusted analyses were not performed. Subgroup analyses may include the CACE analysis previously noted.		
**18-i) Subgroup analysis of comparing only users**		
THE CACE analysis takes into account only data from compliers in the intervention group and would-be compliers in the control group. No other analyses involving only users are included.		
**19) CONSORT**		
These did not occur.		
**19-i) Include privacy breaches, technical problems**		
Privacy breaches and significant techinical problems did not occur during the course of the trial.		
**19-ii) Include qualitative feedback from participants or observations from staff/researchers**		
Qualitative feedback on each module was collected from participants in the experimental group. A summary of this is available from the first author upon request.		
DISCUSSION		
**20-i) Typical limitations in ehealth trials**		
The limitations section addresses potential biases due to attrition, the intervention context, assessment methods, and follow-up duration.		
**21-i) Generalizability to other populations**		
"Thus, it should be taken into consideration that findings of the current study may differ if the intervention program were to be used by non help-seeking cannabis users. Future studies could examine whether online cannabis intervention programs have a significant impact on non treatment-seekers."		
**21-ii) Discuss if there were elements in the RCT that would be different in a routine application setting**		
"The intervention was designed to be fully self-guided, thus requiring minimal therapist input beyond the initial design of the program."<br><br>"This is an effectiveness study, designed to precisely estimate effects that may be obtained in real-world use outside of a research environment. These points engender confidence that the program will have positive effects as a free and publicly available cannabis treatment option."<br><br>The only differences from real-world use involve completion of screening and assessments, all of which are done over the Internet.		
**22-i) Restate study questions and summarize the answers suggested by the data, starting with primary outcomes and process outcomes (use)**		
The Discussion opens by discussing the research hypotheses. A process analysis is also included in this section.		
**22-ii) Highlight unanswered new questions, suggest future research**		
This is addressed in various places in the last seven paragraphs of the paper.		
Other information		
**23) CONSORT**		
ACTRN12609000856213, Australian New Zealand Clinical Trials Registry.		
**24) CONSORT**		
The trial protocol can be accessed from the first author upon request.		

**25) CONSORT**		
"This research was funded by the Department of Health and Ageing (DoHA). DoHA had no role in the development and evaluation of the intervention program."		
**X26-i) Comment on ethics committee approval**		
"Ethical approval for this study was given by the University of New South Wales (UNSW) Human Research Ethics Committee. Approval was granted to recruit participants both within Australia and elsewhere."		
**x26-ii) Outline informed consent procedures**		
"Interested individuals contacted the research team via email and were sent screening and study information materials by return email."<br><br>"Participants were informed that they would be randomly assigned to receive six modules of CBT or six modules of educational information."<br><br>"All participants were given a username and password protected access to their respective websites."<br><br>Upon clicking this link, entry to this website occurred via checking an informed consent box and completion of the baseline assessment questionnaire."		
**X26-iii) Safety and security procedures**		
Data were stored on a secure server to ensure privacy. To reduce likelihood of any harm, participants were provided with contact numbers should they be experiencing significant distress. Additionally, participants diagnosed with a serious mental illness were not included in the study.		
**X27-i) State the relation of the study team towards the system being evaluated**		
"In response to the absence of evidence-based fully self-guided online treatments for cannabis use, the authors developed Reduce Your Use: How to Break the Cannabis Habit."<br><br>"This research was funded by the Department of Health and Ageing (DoHA). DoHA had no role in the development and evaluation of the intervention program."		
